# Teaching Billing and Coding to Medical Students: A Pilot Study

**DOI:** 10.3402/meo.v18i0.21455

**Published:** 2013-08-12

**Authors:** Jiaxin Tran, David Cennimo, Sophia Chen, Eric L. Altschuler

**Affiliations:** 1 Department of Physical Medicine and Rehabilitation, Rutgers New Jersey Medical School, Newark, NJ, USA; 2 Department of Medicine and Pediatrics, Rutgers New Jersey Medical School, Newark, NJ, USA; 3 Department of Pediatrics, Rutgers New Jersey Medical School, Newark, NJ, USA

**Keywords:** billing and coding, evaluation and management services, compliance, education, new curricular material

## Abstract

Complex billing practices cost the US healthcare system billions of dollars annually. Coding for outpatient office visits [known as Evaluation & Management (E&M) services] is commonly particularly fraught with errors. The best way to insure proper billing and coding by practicing physicians is to teach this as part of the medical school curriculum. Here, in a pilot study, we show that medical students can learn well the basic principles from lectures. This approach is easy to implement into a medical school curriculum.

Complex billing practices cost the US health care system billions of dollars annually (1, 2). Coding for outpatient office visits [known as Evaluation & Management (E&M) services] is notoriously poor – with several Medicare audits of outpatient claim records reporting error rates as high as 91% (3). While the new generation of physicians is increasingly cognizant of gaps in their knowledge of proper documentation and coding (4), the existing medical school curricula have largely failed to address this deficit (5). This is unfortunate, as proper practices are best taught early, before bad habits form – making undergraduate medical training the logical place to introduce students proper coding and documentation. Towards this end, we have initiated a programme at our medical school to teach billing and coding to medical students, starting with the basic principles in second year and subsequent applications in later years.

After approaching directors of our school's second-year clinical skills course about teaching billing and coding, it was agreed that this represented an appropriate placement of such materials. Other faculties and the administration were supportive of the idea, and the addition of billing and coding was approved by our school's curriculum committees and, later, by the faculty council.

The objectives for students were to learn the basic principles of billing and coding and, specifically, to be able to successfully bill for outpatient office visits (E&M services). The lecture material was to be reinforced by the evaluation of write-ups and notes done during the second-year clinical skills course and, it was hoped, notes written during the third and fourth years also.

We prepared four 1-hour lectures on E&M billing and coding (see Supplementary Materials online) on the three aspects of billing and coding for E&M visits: (1) medical history; (2) physical examination; and (3) medical decision-making – along with a review lecture. Our initial plan was to give the first two lectures in the fall and the remaining two near the end of second year. However, due to Hurricane Sandy, the fall lectures were postponed until near the end of the clinical skills course.

While student's comments regarding associated lectures were generally positive, reflecting an interest in the topic, the short- and long-term effectiveness of our efforts remains to be rigorously established. In terms of learning outcomes, for example, simple knowledge recall of key elements may be validly assessed via ‘traditional’ exams containing single best answer, multiple choice questions (MCQs). However, measuring more advanced skills or knowledge application – such as those involved in the ‘art’ of actual coding – may require more complex formats, such as mock billing scenarios. Moreover, identifying a level of ‘competent’ performance expected of second-year students will likely also pose a challenge.

Incorporating ‘medical administrative’ content into already over-crowded undergraduate curricula is not without potential concerns. For example, completing regulatory (and other) paperwork needed to comply with current practice requirements is a primary driver of health care costs – making our health care delivery system among the most expensive of any industrialised nation. Arguably, for physicians, these tasks also constitute one of the least desirable aspects of modern medicine. As a result, exposing students early on to the underlying bureaucracy of medical practice could conceivably hasten documented declines in empathy across the undergraduate curriculum.

However, ensuring optimal and timely reimbursement vis-à-vis proper billing and coding is a necessary part of practicing medicine in a capitalist economy. Thus, given the small amount of curricular time needed – combined with encouraging student interest and ‘uptake’ of related material – we believe that integrating some content on billing and coding during undergraduate training may prove, in the long run, to be a beneficial skill.
